# 2482 Reward-based learning as a function of the severity of substance abuse risk in drug-naïve youth

**DOI:** 10.1017/cts.2018.116

**Published:** 2018-11-21

**Authors:** Muhammad A. Parvaz, Kristen Kim, Sean Froudist-Walsh, Jeffrey H. Newcorn, Iliyan Ivanov

**Affiliations:** Icahn School of Medicine at Mount Sinai

## Abstract

OBJECTIVES/SPECIFIC AIMS: Deficits in reward-based learning have been shown in youth at risk for developing substance use disorders (SUD). Here, we investigated whether computational models can be used to more precisely delineate the additive effects of such risk loading (i.e., the comparison between youth with ADHD, and those with ADHD and familial SUD) on reward-based learning in youth. METHODS/STUDY POPULATION: In total, 41 drug-naïve youth, stratified into 3 groups based on ADHD diagnosis and parental SUD: healthy controls (HC, n=13; neither ADHD nor parental SUD), low risk (LR, n=13; ADHD only), and high risk (HR, n=15; both ADHD and parental SUD), performed a reward task. Learning rates, prediction and congruence *t*-scores were computed using a reinforcement learning model and analyzed via a multivariate ANOVA. RESULTS/ANTICIPATED RESULTS: The analyses showed a significant linear effect in task accuracy, which decreased with increasing risk profiles. Analyses of the model-derived variables also showed similar significant linear effects in learning rates and the congruence *t*-score, but not in the prediction *t*-score. These effects were primarily driven by significantly higher learning rate and congruence *t*-score in HC compared with HR youth. DISCUSSION/SIGNIFICANCE OF IMPACT: These results show most profound deficits in reward-learning in HR youth. These findings also show that computational analyses can offer added value over conventional behavioral analyses by more precisely evaluating group differences in relation to SUD risk.

